# The National Physique

**Published:** 1920-08-14

**Authors:** 


### The National Physique.

### INFLUENCE OF THE BOY SCOUT.

At first sight the Boy Scouts' international "Jamboree" at Olympia may seem to have but a remote bearing on that section of The Hospital which is devoted to questions of public health. In reality, however, the movement of which that delightful spectacle was the joyous apotheosis is already having consequences that may well be far-reaching on the life of the nation.

It is a commonplace to say that we are essentially an industrial people, that existence tends more and more to concentrate in the great manufacturing centres. But it was not until Professor Keith published his alarming statistics as to the low average standard of physique of British manhood that the dangerous effects of this dull commonplace came to be generally realised. Professor Keith pointed the only way in which those effects can in the long run be counteracted. The nation must have breathing space. Work in the cities must alternate with recreation in the country. There must be systematic individual and organised exercise. To play games must be as essential a part of life as to eat one's breakfast. That was the clarion call. All over the country the newspapers and the periodicals took up the cry. It was a nine days' wonder. "We must wake up," said everybody in the Press, on the

**THE PUBLIC WELL-BEING—(*continued*).**

platform, and in the pulpit. But after the usual nine days some other wonder took its place, and nothing was done.

But if most of the adults went to sleep, the boys and girls and a few of those determined and unobtrusive social reformers who understand at which end of the scale to tackle the question of human reconstruction remained very wide awake. Scoutmasters and scouts are not very familiar, probably, with Professor Keith's statistics, but they are doing more than any other section of the community to change their significance. They are the sponsors and exemplars of the open-air movement. Go into whatever town or village you will, and you will see these sturdy scout troops marching out into the country for the week-end, playing all sorts of real boys' games, doing just the kind of thing that brings hardihood to the physical frame and joy to the spirit and that breeds an early unconquerable love of trees, flowers, birds, and all the life of the open air. They are pictures of health and good-humour, and the slum boy who is also a scout is as easily distinguishable from the slum boy who is only a slum boy as a white man is distinguishable from a black man.

We are glad to see that the Scout Army is rapidl[illegible] increasing in numbers and extending its influence t[illegible] all parts of the world—civilised and uncivilised. I[illegible] is making an imaginative appeal, in particular, t[illegible] the youth of this country; so much so that, i[illegible] whatever social class a boy may be, not to be [illegible] scout is to be distinctly out of it. The movement[illegible] too, seems now to be in good hands. It is devoid o[illegible] any taint of incipient militarism, in spite of—perhap[illegible] because of—the fact that large numbers of scout[illegible] masters are demobilised officers and men of th[illegible] British Army. It is true, as the "Jamboree[illegible] demonstrated, that they learn to do many thing[illegible] that a soldier does, but then it is their practice t[illegible] learn something from practically every known trad[illegible] and profession of modern times. Their manual skil[illegible] their versatility, and their general "handiness[illegible] were revealed in a remarkable way at Olympia; bu[illegible] what perhaps impressed the onlooker most of a[illegible] was the fine manly appearance of these boys [illegible] many races, their courtesy, their quick intelligence[illegible] and their enthusiasm and vivid capacity for enjo[illegible]ment. A visit to the "Jamboree" would have bee[illegible] excellent medicine for those persons with pess[illegible]mistic views as to the future of the human specie[illegible] In all sincerity we wish the Boy Scouts well.

